# Towards a Unified Understanding of Lithium Action in Basic Biology and its Significance for Applied Biology

**DOI:** 10.1007/s00232-017-9998-2

**Published:** 2017-11-10

**Authors:** Eric Jakobsson, Orlando Argüello-Miranda, See-Wing Chiu, Zeeshan Fazal, James Kruczek, Santiago Nunez-Corrales, Sagar Pandit, Laura Pritchet

**Affiliations:** 1 Beckman Institute for Advanced Science and Technology, University of Illinois at Urbana-Champaign, Urbana, IL USA; 2 Center for Biophysics and Computational Biology, University of Illinois at Urbana-Champaign, Urbana, IL USA; 3 Department of Biosciences, COMSATS Institute of Information Technology, Islamabad, Pakistan; 4 Department of Animal Sciences, University of Illinois at Urbana-Champaign, Urbana, IL USA; 5 Carl R. Woese Institute for Genomic Biology, University of Illinois at Urbana-Champaign, Urbana, IL USA; 6 Neuroscience Program, University of Illinois at Urbana-Champaign, Urbana, IL USA; 7 National Center for Supercomputing Applications, University of Illinois at Urbana-Champaign, Urbana, IL USA; 8 Department of Molecular and Integrative Physiology, University of Illinois at Urbana-Champaign, Urbana, IL USA; 9 Department of Cell Biology, UT Southwestern Medical Center, Dallas, TX USA; 10 Department of Physics, University of South Florida, Tampa, FL USA; 11 Illinois Informatics Institute, University of Illinois at Urbana-Champaign, Urbana, IL USA; 12 Present Address: Department of Psychological and Brain Sciences, University of California at Santa Barbara, Santa Barbara, CA USA

**Keywords:** Ion channels and transporters, Magnesium-dependent enzymes, Physical properties of biological membranes

## Abstract

Lithium has literally been everywhere forever, since it is one of the three elements created in the Big Bang. Lithium concentration in rocks, soil, and fresh water is highly variable from place to place, and has varied widely in specific regions over evolutionary and geologic time. The biological effects of lithium are many and varied. Based on experiments in which animals are deprived of lithium, lithium is an essential nutrient. At the other extreme, at lithium ingestion sufficient to raise blood concentration significantly over 1 mM/, lithium is acutely toxic. There is no consensus regarding optimum levels of lithium intake for populations or individuals—with the single exception that lithium is a generally accepted first-line therapy for bipolar disorder, and specific dosage guidelines for sufferers of that condition are generally agreed on. Epidemiological evidence correlating various markers of social dysfunction and disease vs. lithium level in drinking water suggest benefits of moderately elevated lithium compared to average levels of lithium intake. In contrast to other biologically significant ions, lithium is unusual in not having its concentration in fluids of multicellular animals closely regulated. For hydrogen ions, sodium ions, potassium ions, calcium ions, chloride ions, and magnesium ions, blood and extracellular fluid concentrations are closely and necessarily regulated by systems of highly selective channels, and primary and secondary active transporters. Lithium, while having strong biological activity, is tolerated over body fluid concentrations ranging over many orders of magnitude. The lack of biological regulation of lithium appears due to lack of lithium-specific binding sites and selectivity filters. Rather lithium exerts its myriad physiological and biochemical effects by competing for macromolecular sites that are relatively specific for other cations, most especially for sodium and magnesium. This review will consider what is known about the nature of this competition and suggest using and extending this knowledge towards the goal of a unified understanding of lithium in biology and the application of that understanding in medicine and nutrition.

**Electronic supplementary material:**

The online version of this article (10.1007/s00232-017-9998-2) contains supplementary material, which is available to authorized users.

## Organization of this Paper

We will initially describe the origins of lithium and cite the evidence that lithium is readily transported across membranes but not closely regulated in body fluids. We will further note the absence of evidence for specific lithium transporters or channels, or as a specific cofactor for biological molecules. Evidence for transport and for other biological effects, combined with lack of evidence for specificity, implies that lithium exerts its effects by competing with regulated cations for permeation and binding sites. We will then review and discuss literature describing lithium competitive association and its biological significance in the following contexts:

- Origins of lithium in the environment
- Physical similarities and differences between lithium and other biologically important cations
- Significance and mechanisms for lithium passage across biological membranes
- Lithium interaction with membranes
- Lithium competition with magnesium
- Lithium and mitochondria
- Lithium as a nutrient and a toxin
- Lithium and affective disorders
- Lithium, GSK3, and BDNF**—**Connecting the dots between lithium, stress, affective disorders, and neurodegenerative disease
- Lithium and cancer
- Lithium in the model eukaryote Yeast
- Origins and evolution of lithium in biology

Because of the broad scope of this review, the citation list will be representative rather than exhaustive.

A note on terminology: All biological activity that we know of, and is described in this paper and the cited papers, is with the ion Li+. It has become common practice in the biological literature to use the word “lithium” when describing the biological activity of Li+, with the ionic form understood in the context of the biology. In this review, we adopt this practice except when citing particular papers that use the Li+ notation.

## Origins of Lithium in the Environment

Lithium was one of the three elements produced in the initial condensation of matter from energy immediately following the Big Bang (Fields and Olive 2006). It is widely and unevenly distributed across the surface of the earth in both igneous and sedimentary rock (Turekian and Wedepohl 1961). Its concentration in water is governed by a balance between weathering from rocks into waterways and deposition from water into sediments. Seawater is well mixed with respect to lithium; the concentration is relatively uniform at an average of approximately .025 mM (Schwochau 1984). Lithium levels in freshwater are lower than in seawater and highly variable from location to location. For example, in well water in the United States, lithium concentrations vary from less than .00014 mM to greater than .007 mM (Ayotte et al. 2011). This variability is due to the variations from place to place and throughout evolutionary and geologic time of weathering processes that began shortly after the formation of the earth (Ushikubo et al. 2008). It is from water that lithium moves into living systems, by mechanisms that will be reviewed in this paper.

## Physical Similarities and Differences Between Lithium and Other Biologically Important Cations

Li+ is a monovalent cation. Among the monovalent cations, lithium is the smallest, and sodium the next smallest. Naively, one would expect lithium to compete better for access to sodium sites and transport mechanisms than those of other univalent cations, and this is indeed true, as will be seen in the literature reviewed in this paper. Comparing lithium to divalent cations, the ionic radius of lithium is almost the same as magnesium. Naively, one might expect lithium to compete better with magnesium than with other divalent cations, and this is indeed true as will be reviewed in this paper. In fact, it appears that most of the major biological effects of lithium can plausibly be explained as competition with sodium and magnesium, the ions most like lithium by measures of charge and size. In detail, the underlying physics is associated with the energetic cost for each ion of exchanging its bulk aqueous environment for the complex hybrid aqueous-organic molecule environment of an ion associated with a macromolecule (Varma and Rempe 2008). While the general principles of such selectivity are understood, applying those principles to any case is far from trivial, involving detailed knowledge of the structures of ion-selective biomolecules. A notable paper reported engineering the transformation of a sodium-calcium antiporter into a sodium/lithium-calcium antiporter (Refaeli et al. 2016). This work may help towards a detailed physical understanding of sodium/lithium competition and selectivity. It should also be noted that lithium has been shown to associate with magnesium-ATP (Briggs et al. 2016). The biological importance of ATP leads one to consider to what extent lithium-ATP association may contribute to the overall biological effects of lithium, but at present the connection is not clear.

## Significance and Mechanisms for Lithium Passage Across Biological Membranes

de Roos et al. showed that over a wide range of lithium oral ingestion, lithium levels in blood plasma are almost directly proportional to lithium ingestion (de Roos et al. 2001). This property enables nutrition researchers to use a lithium marker to monitor compliance with controlled diets (Donahoo et al. 2004). From these gross observations it can be inferred that lithium is transported, but that its concentration is not regulated. The lack of regulation is in sharp contrast to other biologically important cations–sodium, potassium, calcium, protons, and magnesium—whose blood concentration is closely regulated. The mechanisms of such regulation are largely (although not totally) understood, and are described in standard textbooks. These mechanisms involve ion permeation and transport mechanisms that are highly specific for the ions being regulated. To date, no transport mechanisms specific for lithium have been identified. On the other hand, there are abundant reports of lithium competition for access to transport mechanisms and binding sites that are otherwise largely specific to closely regulated ions.

One major pathway for lithium entry into cells is through sodium channels, of the voltage-gated class (Richelson 1977), and also of the non-voltage-gated epithelial class (Thomsen and Shirley 2006). A detailed structure and function study of a prokaryotic sodium channel that has strong homology to eukaryotic voltage-gated sodium channels, revealed approximately equal permeability of lithium and sodium (Naylor et al. 2016). Lithium reabsorption in the kidney appears to be largely by its ability to utilize the sodium-phosphate cotransporter in the nephron proximal tubule (Uwai et al. 2014). Without this mechanism, lithium concentrations in the body would be much lower than they in fact are. Lithium substitution for sodium in the environment of a sodium-aspartate cotransporter drives aspartate transport, albeit much less effectively than sodium (Boudker et al. 2007). Both lithium and sodium enter potassium channels, but apparently block rather than permeate (Thompson et al. 2009). Lithium also has some ability to enter calcium channels and inhibit calcium permeation (Kuo and Hess 1993). However, for both potassium and calcium channels, significant inhibition of the channel ionic flux by lithium is achieved only at concentrations far exceeding what can be achieved in living systems. In this case, the value of this knowledge is biophysical, using lithium as a probe for the details of permeation mechanism, rather than understanding the role of lithium in the living system.

In some cases, lithium substitutes for sodium weakly or not at all. One example is sodium-activated potassium channels, for which lithium is a significantly less effective activator than sodium (Kaczmarek 2013). Also, lithium does not effectively replace sodium in sodium-glucose cotransport in the gut epithelium (Csaky 1961).

Lithium efflux from cells is also sodium dependent, as evidenced by observations that lower concentrations of extracellular sodium cause a reduction in lithium efflux in red blood cells (Canessa et al. 1980) and neurons (Szentistvanyi et al. 1979). Experiments on lymphoid cells in which both intracellular and extracellular concentrations are varied suggest that one component of the sodium-dependent lithium efflux is from a sodium-lithium countertransport system in which both sodium and lithium can go both ways (Yurinskaya et al. 2014). The existence of such a system is further supported by a computational model incorporating such a system to explain the data (Vereninov et al. 2016). However, to date, no gene has been identified corresponding to this countertransport. A major mechanism for lithium efflux, for which the gene has been identified, is the sodium-proton pump (Busch et al. 1995). In this case, intracellular lithium competes with protons for access to the pump for ejection from the cell. The efflux of lithium occurs as an example of a general class of secondary active transport mechanisms that are driven by sodium gradients (Krishnamurthy et al. 2009).

Furthermore, lithium also permeates pentameric ligand-gated cation channels (Lewis and Stevens 1983; Grosman, Claudio 2016. Personal communication based on unpublished observations).

In addition to epithelial and nonepithelial cell membranes, lithium transport across mitochondrial membranes is significant. Lithium provides protection against damage to brain mitochondria exposed to elevated calcium (Shalbuyeva et al. 2007). It is plausible that this lithium-dependent protection is mediated by the mitochondrial Na+/Ca++ exchanger (NCLX), whose primary function is to mediate sodium-calcium exchange across the mitochondrial outer membrane, and is also an efficient lithium-calcium exchanger (Boyman et al. 2013). There are many other ion transport mechanisms across the mitochondria outer and inner membranes that are potential pathways for lithium transport (Szabo and Zoratti 2014) but their role in transporting lithium has not been reported.

Because the biomolecules that move lithium across membranes in multicellular animals have homologues in prokaryotes (BLAST search, unpublished), it appears that living systems have taken up lithium from their surroundings from very early in evolution.

**Fig. 1:**
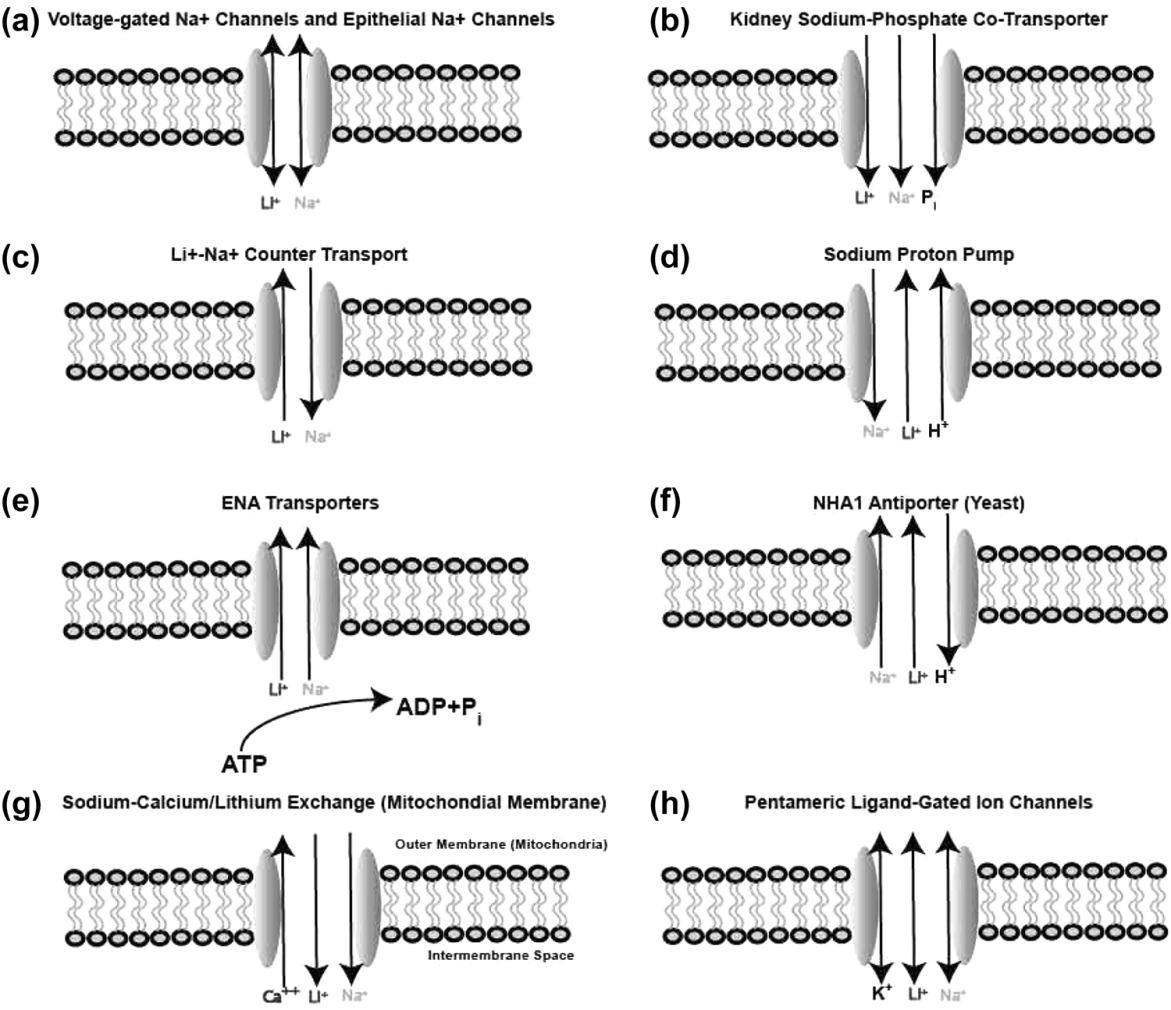
Major mechanisms by which lithium crosses biological membranes. **a** Lithium permeates both voltage-gated (Richelson 1977; Naylor et al. 2016) and epithelial (Thomsen and Shirley 2006) channels that are otherwise selective for sodium. **b** Lithium is reabsorbed in the kidney by the sodium-phosphate cotransporter (Uwai et al. 2014). **c** A major mechanism for lithium efflux from cells is a sodium-lithium countertransport (Szentistvanyi et al. 1979; Yurinskaya et al. 2014; Vereninov et al. 2016). **d** Another significant lithium efflux mechanism is the sodium-proton pump (Busch et al. 1995). **e** Members in yeast of the ENA family of cation-transport ATPases move lithium as well as sodium (Ruiz and Arino 2007). Based on sequence homology, we hypothesize that the mammalian members of the family will share this functional feature. **f** The NHA1 antiporter in yeast is important for both sodium and lithium efflux (Prior et al. 1996). This antiporter does not appear to have mammalian orthologs. **g** Lithium protects mitochondria against the effects of elevated calcium (Shalbuyeva et al. 2007), presumably through its action in the sodium-calcium antiporter in the mitochondrial outer membrane (Boyman et al. 2013). **h** Lithium permeates pentameric ligand-gated ion channels (Grosman, Claudio 2016. Personal communication based on unpublished observations; Lewis and Stevens 1983)

Intracellular lithium concentrations are seldom measured, but one study found that the ratio of intracellular to extracellular lithium concentrations in red blood cells is approximately one-half (Mendels and Frazer 1973). Major mechanisms by which lithium crosses membranes, and which, therefore, account for the transmembrane distribution, are summarized graphically in Fig. 1.

## Lithium Interaction with Membranes

**Fig. 2:**
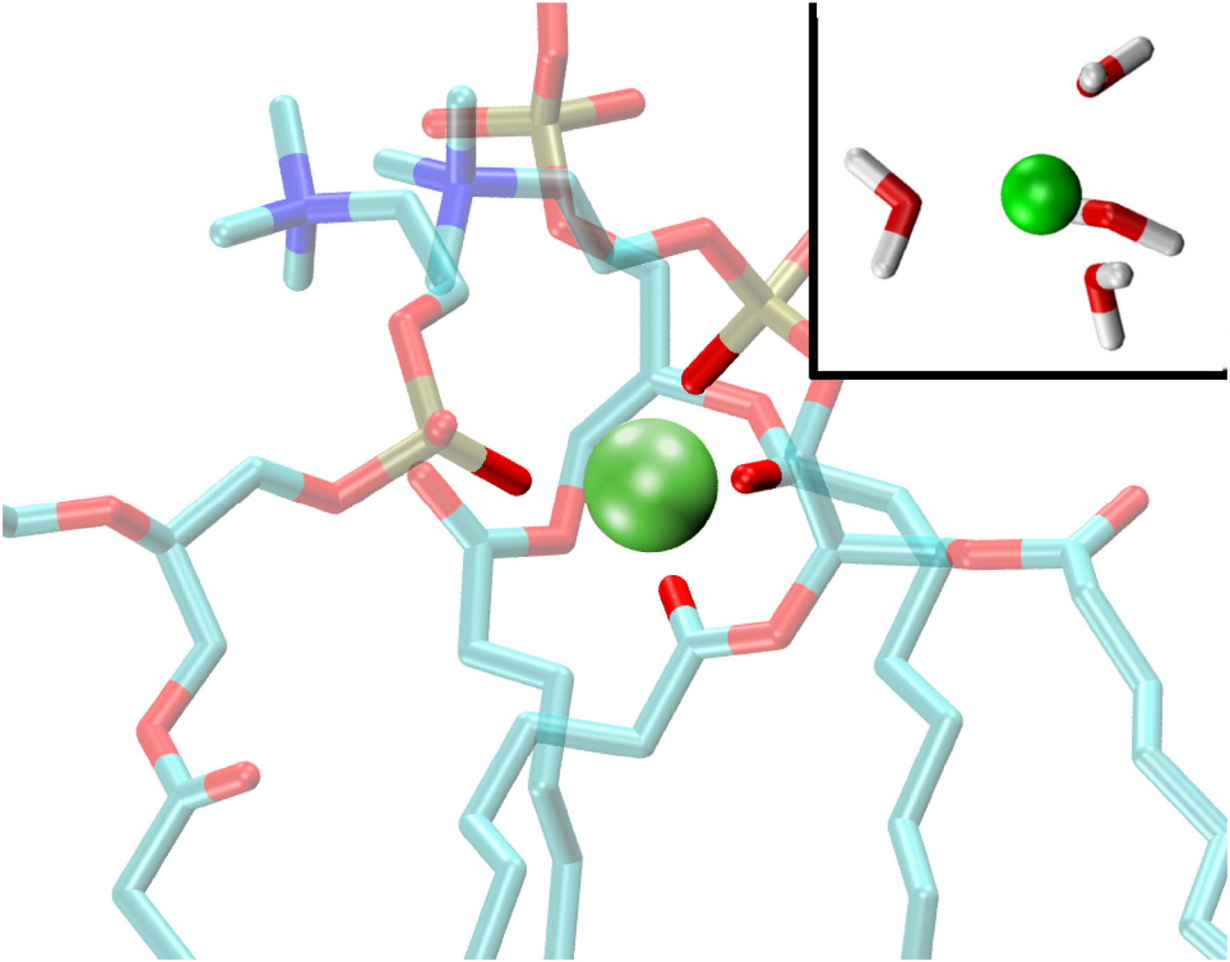
Snapshot of a common conformation observed in molecular dynamics simulations of lithium ion coming out of aqueous solution and interacting with a POPC membrane (Kruczek et al. 2017). The lithium ion has induced formation of a tetrahedral shell involving a phosphate group and an sn-2 carbonyl group from one phospholipid molecule plus an sn-2 carbonyl from a second phospholipid and a phosphate group from a third phospholipid. Larger alkali metal ions such as sodium or potassium are found to induce this structure only rarely or not at all. The tetrahedral cage geometry in the membrane mimics the tetrahedral geometry of the first shell of hydration waters for the lithium ion in solution (seen in upper right). While lithium commonly has a four-fold coordination in its inner hydration shell, sodium and potassium have six and seven, respectively (Mähler and Persson 2011)

Published information about the interaction of lithium with membranes is fragmentary but suggests that such interactions are biologically significant. A deuterium NMR study explored the ability of potassium, sodium, lithium, magnesium, and calcium ions to alter the structure of 1-palmitoyl-2-oleoyl-3-glycero-phosphatidylserine (POPS) and (1-palmitoyl-2-oleoyl-3-glycero-phosphatidylcholine (POPC) membranes (Roux and Bloom 1990). This study found that lithium penetrated the membranes more deeply, and altered membrane structure to a greater degree, than sodium or potassium but not quite as much as the divalent cations. The effect of ions on modulating the diffusivity of hydration water near the surface of a POPC vesicle was explored using Overhauser dynamic nuclear polarization (Song et al. 2014). It was found that sodium and potassium increased the activation energy for water motion, while lithium and calcium reduced such energy. In another study, microelectrophoresis was used to determine the degree of association of different alkali metal ions with phosphatidylcholine (PC) membranes (Kotyńska et al. 2016). It was found lithium had the highest overall affinity for the membranes. Studies based on zeta potential find that lithium has the highest affinity of all alkali metal ions for a mixed PC-PG membrane (Maity et al. 2016). The ordering of ionic affinities as a function of counterions and of headgroups can be ordered into a Hofmeister-like series (Vlachy et al. 2009). A computational study of alkali metal ions and POPC membranes confirmed the high affinity of lithium and showed that lithium-induced conformational changes in the head groups that were not seen with the other ions (Kruczek et al. 2017). The nature of the conformational change is illustrated in Fig. 2. This figure shows a snapshot of a typical situation in the above-cited molecular dynamic simulations in which a lithium ion has penetrated the interior of a POPC bilayer and induced a tetrahedral cage structure involving four negatively charged groups from three different phospholipid molecules. This geometry is like the tetrahedral structure formed by water molecules around the lithium ion in solution. The larger alkali metal ions can’t readily form these tetrahedral structures in the membrane and have larger coordination numbers than four for their inner hydration shell in solution (Mähler and Persson 2011).

Lithium was reported to compete with both calcium and magnesium for specific sites in phosphatidylcholine-phosphatidylglycerol membranes (Fossel et al. 1985).

In biological membranes of mixed composition, it was found that lithium treatment of experimental animals altered the fluidity of neural plasma membrane (Lopez-Corcuera et al. 1988). The changed fluidity appeared to be not due to direct interaction of the lithium with the membrane but rather to metabolic effects of lithium that, over course of the lithium treatment, altered the saturation of the lipid chains in the membrane. A more recent study utilizing C6 glioma cells indicates that the distribution of the signal protein GS alpha is moved into lipid rafts by lithium (Donati et al. 2015). It is not clear whether this reorganization of the membrane was due to direct effect of lithium or due to lithium-induced metabolic changes that modified the membrane composition.

## Lithium Competition with Magnesium

It has long been known that in rocks and clays of all ages and across the world, lithium and magnesium readily substitute for each other (Goldschmidt 1937). This is attributed to the fact that their ionic radii are almost identical to each other, and quite different from the ionic radii of other plentiful monovalent and divalent ions. With a similar size, we might expect lithium and magnesium to compete for the same binding sites in biomolecules, despite the different magnitude of their charge. In early work, Birch noted the systematic tendency of lithium to inhibit magnesium-dependent enzymes (Birch 1974). We now have many more published examples of such competition. Lithium appears to inhibit β-adrenergic and muscarinic receptor coupling to G proteins by competing with magnesium, which facilitates such coupling (Avissar et al. 1991; de Mota de Freitas et al. 2006; Yoshikawa and Honma 2016; Bauer and Gitlin 2016; Wang and Friedman 1999). A particularly important example is substitution of a lithium ion for a magnesium ion acting as a cofactor in inositol monophosphatase, a putative critical molecular target for lithium therapy for bipolar disease (Singh et al. 2013). In this protein, the binding site for lithium is not revealed in crystallography nor in solution NMR but can be identified in magic angle spinning solid state NMR, which is more suitable for systems with large internal motion (Haimovich et al. 2012). Another target of lithium, also a magnesium-dependent phosphatase and with relevance to neural effects, is bisphosphate 3-prime-nucleotidase (BPNT1) (Spiegelberg et al. 2005; Meisel and Kim 2016). These findings are consistent with a hypothesis that lithium inhibits magnesium-dependent enzymes by displacing magnesium from its binding site thereby reducing the structural stability and lowering activity of the enzyme.

One mode of action with many consequences is lithium inhibition of glycogen synthase kinase 3beta (GSK3B), initially shown in vitro and in intact cells (Stambolic et al. 1996), and in the context of embryonic development (Klein and Melton 1996). It was later shown that lithium exerted its inhibitory effect on GSK3B by competing with magnesium for an essential binding site (Ryves and Harwood 2001). There are two closely related forms of GSK3, termed alpha (GSK3A) and beta (GSK3B), which are equivalently inhibited by lithium (Freland and Beaulieu 2012). The two forms of GSK3 have substantial functional redundancy (Doble et al. 2007). However, some of their physiological properties are different, as demonstrated by the fact that GSK3B knockout mice are not viable (Hoeflich et al. 2000), but GSK3A knockout mice survive (MacAulay et al. 2007). The very widespread nature of GSK3B effects is related to the large number of transcription factors that it regulates (Grimes and Jope 2001). It functionally modulates cellular threshold for apoptosis (Beurel and Jope 2006), it is central to mediating mitochondrial response to stress; (Juhaszova et al. 2004) it facilitates immune responses by enabling the nuclear export of NF-ATc; (Beals et al. 1997) it regulates inflammation; (Beurel et al. 2010) it regulates cardiac hypertrophy and development (Hardt and Sadoshima 2002), to name just a few. Based on microarray studies of brain cells in animals, lithium alters gene expression patterns significantly (McQuillin et al. 2007), to be expected due to the large number of transcription factors regulated by GSK3B. Small molecule inhibitors of GSK3 are effective in the micromolar range (Coghlan et al. 2000), while lithium effects are significant only at much higher concentrations. Mice heterozygous for GSK3B exhibit similar behavioral traits to wild type littermates treated with 1 mM lithium (a concentration that inhibits about 25% of GSK3 activity, in line with 1 of the 4 alleles of GSK3 inactivated in the GSK3B heterozygous mice) (O’Brien et al. 2004).

For all of the widely-noted importance of the interaction between GSK3 and lithium, the precise biophysics of this interaction is not completely understood. A complicating factor is that GSK3’s mode of action involves an interaction with ATP. ATP is associated with Mg++ and with Li+, while Mg++ is also an essential cofactor of GSK3. Combining these factors with the propensity of Li+ to compete with Mg++ for ionic binding sites, the full story appears to be sufficiently complicated to pose a significant challenge to current computational and experimental methods of elucidating biomolecular structure and function.

It should be noted that lithium also inhibits the activity of GSK3 by a second method in addition to competition with magnesium, that is, by increasing phosphorylation (Jope 2003). It appears likely that this effect is secondary to direct lithium inhibition of a phosphatase. In addition to inhibiting the activity of GSK3B, lithium also inhibits its transcription (Mendes 2009).

Of all kinases, GSK3 appears to have the largest number of known substrates, over 100 known (Beurel et al. 2015) and about 500 predicted by theory based on scanning and interpreting relevant motif sequences in the human genome (Linding et al. 2007). Lithium will thus to some extent modulate activity along all pathways containing the hundreds of GSK3 substrates. Any comprehensive understanding of lithium action therefore can’t just be a summation of actions on individual molecules, but rather must include genome-wide systems understanding of how the action of lithium, through inhibition of GSK3, modulates the activity of the hundreds of substrates regulated by GSK3.

**Fig. 3:**
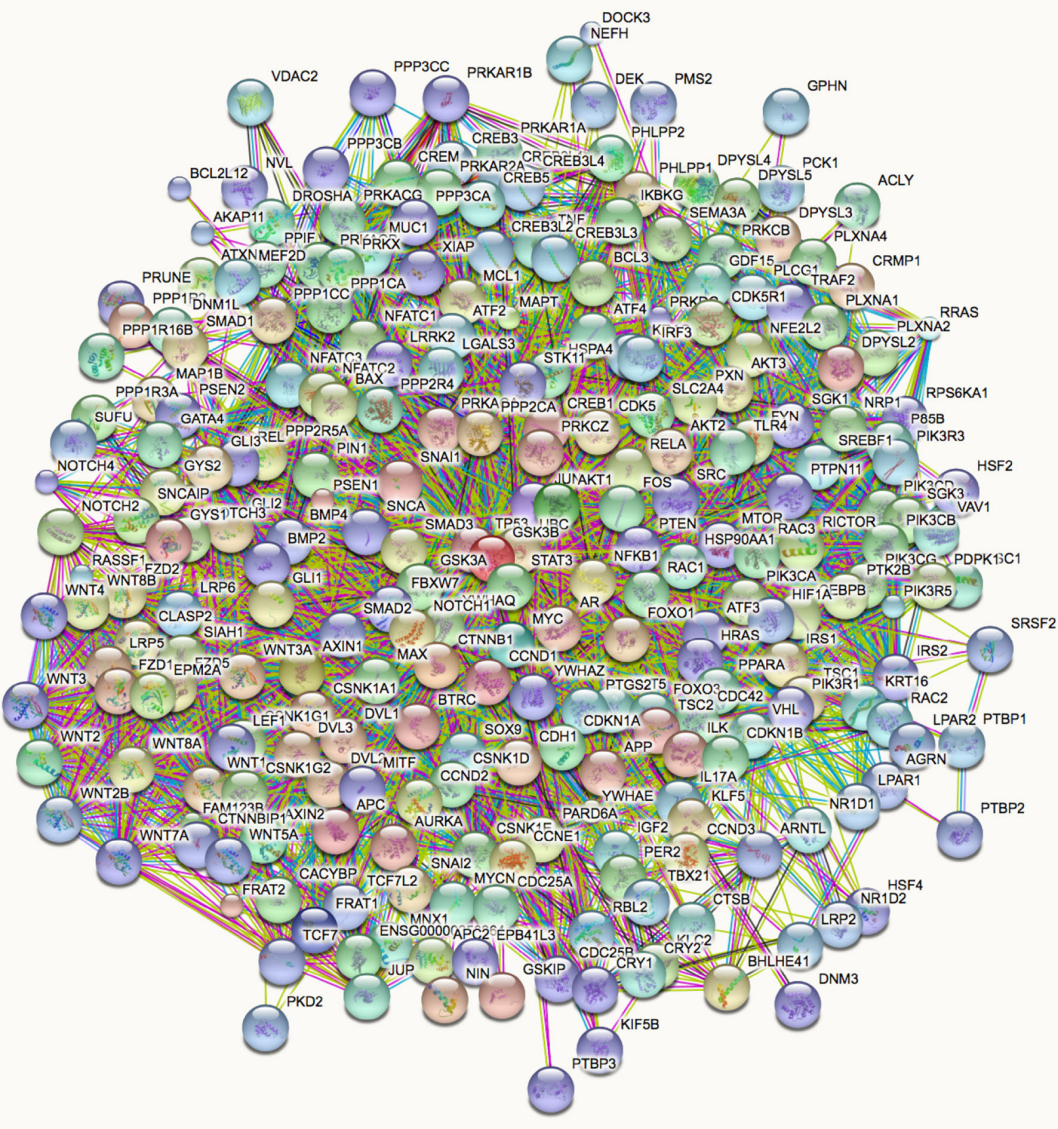
Interaction diagram for GSK3B and the 265 other proteins with which it interacts directly with 70% confidence, based on the STRING protein–protein interaction database. Protocol for replicating the search for an up-to-date version of this network is provided in Supplementary Material

Figure 3 shows graphically 265 proteins that interact directly with GSK3B at a 70% confidence level, as tabulated in the STRING protein–protein interaction database (Szklarczyk et al. 2016). An exact protocol for replicating the search that resulted in this figure and in calculated enriched gene ontology categories and KEGG pathways for this network of genes are given in supplementary material.

Since lithium affects other magnesium-dependent proteins, the output from the STRING search provides a minimal assessment of lithium effects on biological processes and pathways. There are estimated to be over three thousand human proteins with magnesium binding sites (Piovesan et al. 2012).

## Lithium and Mitochondria

Compared to the cytosol, mitochondria contain much higher levels of active (unphosphorylated) GSK3B (Bijur and Jope 2003), suggesting that mitochondrial function may be especially sensitive to lithium levels. There is also substantial evidence that mitochondrial damage and dysfunction are associated with both affective disorders (Rezin et al. 2009; Manji et al. 2012) and neurodegenerative disease (Lin and Flint Beal 2006). Thus, the question: To what extent are effects of lithium on affective disorders and neurodegeneration due to effects of lithium on mitochondrial function?

One relevant study showed that both lithium and the mood stabilizer valproate have protective effects against amphetamine-induced mitochondrial dysfunction in rats (Valvassori et al. 2010; Bachmann et al. 2009). An increase in complex I and complex II activities of the mitochondrial respiratory chain was shown to be induced by lithium at therapeutic concentrations in human cortical brain tissue, thus suggesting a potential of lithium to enhance oxidative phosphorylation (Maurer et al. 2009). A non-invasive study on human bipolar patients found that treatment with lithium reduced plasma levels of oxidative stress markers (Machado-Vieira et al. 2007), consistent with the more invasive studies.

In addition to affective disorders and neurodegeneration, GSK3B in mitochondria is deeply involved in the anti-apoptotic phenotype that characterizes cancer cells (Beurel and Jope 2006; Rasola and Chiara 2013).

## Lithium as a Nutrient and a Toxin

Because of its ubiquity in our environment, ingestion of lithium in at least trace amounts is unavoidable. Based on experiments in which animals are deliberately lithium-deprived and suffer a decline in fertility, lithium is probably an essential trace element in the diet (Anke et al. 1991; Pickett and O’Dell 1992; Schrauzer 2002). At the other extreme, lithium sufficient to raise plasma levels to above approximately 1.2 mM will cause acute toxicity (Malhi et al. 2011) (nausea, diarrhea, extreme trembling). Above 2 mM, death may occur. At levels maintained over years in lithium therapy for bipolar disorder (usually 0.5–0.8 mM), there appear to be statistically significant chronic problems with renal function, thyroid function, and parathyroid function (McKnight et al. 2012). These problems can generally be managed and are far less debilitating than untreated or ineffectively treated bipolar disorder (Malhi and Berk 2012). Based on epidemiological studies, it appears that sub- therapeutic levels of lithium may have a beneficial effect on mood disorders. Multiple independent studies based on populations from different parts of the world have explored the statistical relationship between lithium concentration in drinking and per capita rate of suicide. Most of these studies have found that the more lithium in the drinking water, the lower the suicide rate (Schrauzer and Shrestha 1990; Ohgami et al. 2009; Helbich et al. 2012; Giotakos et al. 2013; Blüml et al. 2013; Kapusta et al. 2011). One study has found an inverse correlation between lithium in the drinking water and homicide rates (Giotakos et al. 2015). More broadly, one study finds an inverse correlation between lithium concentration in drinking water and age adjusted mortality from all causes, even excluding suicide (Zarse et al. 2011). By contrast, some environmental toxicologists seem to view lithium in ground water solely as a pollutant (Aral and Vecchio-Sadus 2008). Indeed, it would be naïve to leap to a conclusion that more lithium in drinking water is either beneficial or harmful in every way, since lithium has so many diverse effects. One possible point of concern is pregnant women, since epidemiological studies have found reduced thyroid function during pregnancy (Harari et al. 2015) and reduced birth weight (Harari et al. 2015), with higher concentrations of lithium in drinking water.

The wide range of lithium concentrations between background from drinking water and food on the one hand, and therapeutic doses for bipolar disorder on the other hand, remain almost totally unexplored. One multi-year study of low dose lithium (lithium blood concentration 0.25–0.5 mM), found no evidence of reduced renal function (Aprahamian et al. 2014).

## Lithium and Affective Disorders

The clinical history of lithium for the treatment of affective disorders began with the 1949 report by John Cade that he had successfully treated ten manic patients with lithium (Cade 1949). Subsequent experiments scaled Cade’s observations to many more subjects (Baastrup and Mogens 1967; Schou 1968). Today, the guidelines of major international psychiatric associations recommend lithium as a first-line therapy for bipolar disease, including the American Psychiatric Association (American Psychiatric Association 2002), The World Federation of Societies of Biological Psychiatry (Grunze et al. 2004), the Canadian Network for Mood and Anxiety Treatments (CANMAT) (Yatham et al. 2013) and the International Society for Bipolar Disorders (ISBD) (Yatham et al. 2013).

The serotonin theory of depression was suggested by animal experiments using tricyclic antidepressants (de Montigny and Aghajanian 1978) and confirmed by lithium animal experiments (Treiser et al. 1981) and clinical experience (Souza and Goodwin 1991). This theory (Price et al. 1990), combined with the goal of greater specificity of action than provided by lithium or other antidepressants, led to Prozac and the family of selective serotonin reuptake inhibitors (SSRI’s)—not only a medical but also a cultural revolution (Kramer 1993). In addition to lithium’s primary role in treating bipolar disorder, it is also useful as an augmentation to other antidepressants in treatment of unipolar depression (Crossley and Bauer 2007; Nelson et al. 2014). Lithium is the most successful among all antidepressants and mood stabilizing drugs in preventing suicide (Bschor 2014; Cipriani et al. 2013).

Experiments have been reported on the efficacy of administering lithium to inhibit aggressive behavior in inmates of penal institutions (Sheard 1971) and residents of psychiatric hospitals (Craft et al. 1987; Campbell et al. 1995). In each case, it was reported that individuals receiving lithium exhibited less aggressive behavior than individuals in a control group.

Progress has been made in identifying the specific genes and pathways underlying affective disorders, and how lithium modulates at the molecular level. One such gene, originally identified by patterns of mutations in human families, is Disrupted in Schizophrenia-1 (DISC1) (Porteous et al. 2011), in turn regulated by Dixdc1 (Singh et al. 2010). Dixdc1-knockout mice demonstrate behaviors interpreted as mouse versions of human affective disorders, which behaviors are reversed by lithium, presumably by lithium’s inhibition of GSK3B (Martin et al. 2016).

## Lithium, GSK3, BDNF—Connecting the Dots Between Lithium, Stress, Affective Disorders, and Neurodegenerative Disease

Reduced serum levels of brain-derived neurotrophic factor (BDNF) have been implicated in depression (Karege et al. 2002), bipolar disorder (Cunha et al. 2006; Post 2007), and dementia (Weinstein et al. 2014). GSK3B inhibits BDNF-induced signaling (Mai et al. 2002). Since lithium inhibits GSK3, it might be expected that lithium would enhance BDNF activity. Indeed, in animal experiments, lithium was shown to enhance the expression of brain-derived BDNF (al 2002). In addition, BDNF has been shown to play an important role in survival of adult and developing central neurons both in culture and in vivo (Berton et al. 2006; Ghosh et al. 1994; Acheson et al. 1995; Conover et al. 1995; Jones et al. 1994). The role of lithium in increasing expression of BDNF plus the role of BDNF in survival of neurons has led to the suggestion that lithium might have a role to play in the treatment of neurodegenerative disease (Chuang 2004).

Several other reported research results have supported the potential of lithium for treatment of neurodegenerative disease, as reviewed by Forlenza et al. (Forlenza et al. 2014). However, relevant clinical trials remain to be done. A major issue is that, because neurodegeneration takes place over several years, the most significant clinical trials would need to involve lithium treatment for a significant number of years, with long-term follow-up of the subjects. The only substantial population of long-term lithium users consists of people who are receiving lithium treatment for bipolar disorder. Not all bipolar patients receive lithium, so one can gather statistics about the incidence of neurodegenerative disease among elderly bipolar patients who have received long-term lithium treatment, and those who have not. In one such study, in an otherwise well-matched cohort of elderly (approximately 70 years old), 5% of those on long-term lithium therapy (continuous for the previous five years) were diagnosed with Alzheimer’s disease (AD), while 33% of those not receiving consistent lithium therapy were diagnosed with AD (Nunes et al. 2007).

Another type of long-term study is epidemiological. A recent nationwide study in Denmark showed that lithium level in the drinking water was significantly correlated with incidence of dementia, with higher lithium levels showing lower levels of dementia (Kessing et al. 2017).

The early stage of frontotemporal dementia (FTD) typically involves behavior resembling an affective disorder (Woolley et al. 2011), posing a challenge for diagnosis. When FTD is suspected, a definitive diagnosis in the early stage of the disease requires neuroimaging (McMillan et al. 2014). It is generally believed that such a diagnosis, when correct, is a death sentence, typically over a period of 2 to 14 years. However, there may be one documented apparent exception to the incurability of FTD, in a case history presented by Monji et al. (2014). In this study a middle-aged man presented affective symptoms that had no apparent origin in early life. Imaging revealed abnormalities typical of FTD. Therefore, a diagnosis of FTD was made. However, because the psychiatric symptoms had a pattern like bipolar disease, lithium therapy was begun. In a little under 2 years the psychiatric symptoms had been completely mitigated and new brain images appeared normal. The authors concluded that the initial diagnosis of FTD was in error. However, the data presented in the paper were also consistent with the hypothesis that the FTD diagnosis was correct and that the lithium therapy reversed the course of the disease. We corresponded with Dr Monji and he agreed via e-mail that this hypothesis is also consistent with the evidence underlying the report from him and his collaborators. If this interpretation is correct, it would provide a counter-indication to the prevailing belief that FTD is invariably fatal. The efficacy of lithium for FTD patients is to be tested in a newly announced clinical trial (https://clinicaltrials.gov/ct2/show/NCT02862), although only with respect to relief of behavioral symptoms over the course of a 12-week trial. The limited scope of this study is a continuation of a line of thought that considers affective and neurodegenerative symptoms of FTD as relatively separate (Huey et al. 2006).

Response to stress is not a disorder but rather an adaptive response to an environmental challenge, mediated by release of glucocorticoids (Sapolsky et al. 2000). However stressful events can also cause maladaptive responses, including major episodes of depression (Hammen 2005). In an animal model for stress-induced depression, the induction of depression was found to be facilitated by activity of GSK3B and blocked by lithium (Silva et al. 2008). One possible mechanism for induction of depression by stress is inhibition of adult hippocampal neurogenesis, in response to glucocorticoids (Snyder et al. 2011). Lithium was found to counter the effects of glucocorticoids on adult hippocampal neurogenesis (Boku et al. 2009).

## Lithium and Cancer

Using lithium, GSK-3 inhibition has been shown specifically to inhibit prostate tumor growth (Mazor et al. 2004).

Table 1 shows the 13 KEGG pathway categories that are most statistically enriched in the 265 genes shown in Fig. 3 that are the direct interactors with GSK3B. This table was generated using the analysis tools built into the STRING protein–protein interaction web site. All of these pathways are implicated in cancer. The Wnt signaling pathway (second on the list) is implicated in both cancer (Anastas and Moon 2013; Duffy et al. 2016) and neurodegenerative disease (De Ferrari et al. 2003). The extremely low false discovery rates show that these statistical enrichments could not have occurred by chance, implying that the lithium effect on these pathways, exerted through its inhibition of GSK3B, indeed has significant implications for cancer incidence and therapy.

**Table 1:** The 13 KEGG pathways most enriched in the 265 genes that interact directly with GSK3B

Pathway ID	Pathway description	Gene count	FDR
05200	Pathways in cancer	77	1.86e−74
04310	Wnt signaling pathway	54	4.78e−65
05166	HTLV-1 infection	65	6.33e−65
04390	Hippo signaling pathway	46	1.4e−49
05205	Proteoglycans in cancer	52	2.36e−49
05161	Hepatitis B	45	2.97e−49
04151	Pl3K-Ak1 signaling pathway	59	2.97e−49
05215	Prostate cancer	38	2.97e−49
05217	Basal cell carcinoma	32	2.97e−49
05203	Viral carcinogenesis	45	1.28e−43
05213	Endometrial cancer	28	5.89e−39
04916	Melanogenesis	34	9.16e−39
05210	Colorectal cancer	29	9.16e−39

All of these pathways are implicated in cancer. The false discovery rates show that the probability that the degrees of enrichment observed could have occurred by chance, is vanishingly small. Protocol for replicating this search in the STRING database is provided in Supplementary Material

The possible use of lithium as an anticancer agent is reinforced by a retrospective study showing that psychiatric patients undergoing lithium therapy for bipolar disorder had a much lower incidence of cancer than a matched group not receiving lithium therapy (Cohen et al. 1998). More recent studies of similar design, one conducted nationwide across Sweden, and another across Taiwan, achieved the same result (Martinsson et al. 2016; Huang et al. 2016). On the other hand another nationwide study, this time from Denmark, showed no correlation of lithium with colorectal adenocarcinoma (Pottegård et al. 2016). On closer look, the Denmark study does not contradict the Swedish study. The Swedish study also found that for the entire population lithium was not correlated with cancer incidence, but in addition found that bipolar individuals not treated with lithium had a higher incidence of cancer than the general population. Lithium-treated bipolar patients, on the other hand, had essentially the same cancer incidence as the general population.

A detailed study of molecular mechanisms by which lithium inhibition of GSK3-beta inhibits proliferation of prostate tumor cells in culture was presented by Sun et al. (Sun et al. 2007). The work was subsequently extended to an animal model (Zhu et al. 2011). A clinical trial for the effect of lithium coupled with prostatectomy on men has been conducted, but results not yet published (https://clinicaltrials.gov/ct2/show/NCT02198).

Lithium has been found to be lethal to neuroblastoma cells but not to normal nerve cells (Duffy et al. 2014). The experimentally determined effective dose was 12 mM, a level which would be lethal if achieved systemically in a human or model organism but perhaps could be induced locally. A similar effect was found in ovarian cancer cells (Cao et al. 2006), although a subsequent similar study on ovarian cancer cells suggests only a more modest benefit (Novetsky et al. 2013). It is not clear from our reading of the two ovarian cancer papers why the results are significantly different from each other.

With respect to other cancers, one study suggests that lithium inhibits proliferation of a colorectal cancer cell line (Li et al. 2014). Another study on colon cancer cells showed that lithium specifically induced a reversal of the epithelial-to-mesenchymal transition characteristic of the cancer cells (Costabile et al. 2015).

Two studies with relatively small sample size suggested a possible link between lithium and tumors of the upper urinary tract (Rookmaaker et al. 2012; Zaidan et al. 2014). However a large-scale study involving all urinary tract cancers in Denmark over a multi-year period found no correlation with lithium use (Pottegård et al. 2015).

Because lithium therapy is systemic rather than topical or local, it follows that lithium might inhibit metastasis. Evidence that this is the case for colon cancer comes from observation of inhibition of metastasis-inducing factors by lithium and by observation on reduced metastasis in model animals given lithium therapy (Maeng et al. 2016).

## Lithium in the Model Eukaryote Yeast

In the belief that yeast would be a useful model eukaryotic organism, the yeast genome sequence was completed in 1996 (Goffeau et al. 1996). This belief has been amply justified in the intervening years, notably by the award of the 2016 Nobel Prize in Medicine to Yoshinori Ohsumi for his discoveries of mechanisms of autophagy, initially conducted in yeast and then extended to humans and other metazoan (Levine and Klionsky 2017). The yeast genome is rich in homologs of the lithium-sensitive mammalian enzymes mentioned earlier in this review. It thus may be that comparative molecular research with yeast and animal cells and organisms can provide new understandings.

An early general description of the effects of lithium on yeast was made by Asensio et al. (Asensio et al. 1976). Different strains have different levels of sensitivity (Petrezselyova et al. 2010). Studies on yeast halotolerance revealed that Met22p/Hal2p, a conserved adenosine 3,5-bisphosphonate phosphatase which can be inhibited by submillimolar concentrations of lithium (IC50 0.1 mM), conferred lithium resistance when overexpressed (Murguia et al. 1995). Once Met22/Hal2 is inactivated, adenosine bisphosphonates accumulate leading to the inhibition of sulfotransferases and RNA processing enzymes such as exoribonuclease Xrn1p (Dichtl et al. 1997). The structure of Hal2p has been determined (al 2000) and found to be structurally related to the human BPntase/RnPIP which presents a similar behavior towards lithium (al 2005). Comparative studies across multiple organisms revealed a similar tertiary structure for several phosphomonoesterases involved in inositol signaling, gluconeogenesis, sulfate assimilation, and quinone metabolism, including an ion-binding consensus sequence (D-Xn-EE-Xn-DP(i/l)D(s/g/a)T-Xn-WD-X11-GG) which is required for catalysis and could be the potential docking site for lithium (York et al. 1995).

Among the family of related phosphomonoesterases, inositol monophosphatase (IMPase) from both yeast and from mammalian brain can be inhibited in vitro at concentrations used to treat bipolar disease (0.5 to 1.0 mM) (Hallcher and Sherman 1980; Lopez et al. 1999). The inactivation of IMPase blocks the production of inositol from inositol monophosphate (IMP), shutting down the biosynthetic pathway that leads to the generation of inositol-1,4,5 trisphosphate (IP3), a central molecule for several signaling cascades (Berridge 2016). A peculiar feature of lithium-caused inositol depletion is a positive feedback mechanism, in which the incremental effect of lithium is greater the higher the concentration of the IMP-IMPase complex (Berridge et al. 1989). Theory of dynamical systems shows that positive feedback loops create bistable or multistable states (Cinquin and Demongeot 2002). The dramatic and persistent affect shifts in bipolar disorder, and the dramatic change upon treatment with lithium (https://www.nimh.nih.gov/health/topics/bipolar-disorder/index.shtml), suggest multistable states in the biochemical networks underlying bipolar disorder, possibly related to the inositol depletion positive feedback loop, and other positive feedback loops yet to be identified. The depletion of inositol has been confirmed in organisms as diverse as Xenopus embryos and Dictyostelium (Maslanski et al. 1992; al 1996). However, because of lithium’s diverse targets, for example GSK3 as well as IMPase inhibition, no lithium-induced phenotype may be ascribed to any one of lithium’s molecular actions without specific controls. Alternatives to the “inositol depletion hypothesis” have been presented for lithium’s effects on development (Klein and Melton 1996) and on affective disorders and neurodegenerative disease (Harwood 2005). Because lithium has so many targets, it should be expected in general that no specific modulator of any gene or gene product will completely emulate lithium.

The observation that yeast cells are very sensitive to lithium when grown on galactose, led to the identification of phosphoglucomutase as another lithium target (Masuda et al. 2001). This key enzyme in the sugar metabolism converts glucose-1-phosphate into glucose-6-phosphate. Importantly, yeast (IC50–0.2 mM) and human (IC50–1.5 mM) phosphoglucomutase can be significantly inhibited by lithium in vivo (Csutora et al. 2005). In yeast cells grown on galactose this leads to the accumulation of glucose 1-phosphate and galactose 1-phosphate which impact the balance of trehalose and glycogen, the carbohydrates used for energy storage in yeast (Bro et al. 2003). The overexpression of phosphoglucomutase Pgm2p was shown to rescue lithium sensitivity in galactose medium (Masuda et al. 2001). This discovery shows that some lithium targets can only be revealed by analyzing the behavior of yeast cells in conditions other than growth on rich media. For instance, under starvation conditions, yeast cells trigger a differentiation pathway that leads to meiosis and gametogenesis (van Werven and Amon 2011). Interestingly, it was early noticed that gametogenesis in yeast is more sensitive to lithium than normal proliferation (Asensio et al. 1976). We have reproduced this result (unpublished), but the mechanism responsible for the inhibition of gametogenesis is still not clarified. A similar result has been observed in the slime mold Dictyostelium (Maeda 1970). The yeast homolog of the mammalian lithium-sensitive GSK3B, Rim11p, is a major regulator of meiotic entry (Rubin-Bejerano et al. 2004; Bowdish et al. 1994), and could therefore be a target of lithium during gametogenesis. Due to the number of enzymes upregulated during the meiotic differentiation program, other meiosis-specific proteins could also be lithium sensitive.

In yeast as in mammals, membrane mechanisms that transport sodium also transport lithium (Wieland et al. 1995; Ariño et al. 2010). Yeast extrudes both sodium and lithium through the NHA1 proton-sodium antiporter (Prior et al. 1996) and through the PMR2/ENA family of ATPases (Ruiz and Arino 2007). Experiments comparing lithium sensitivity of haploid vs. diploid yeast cells showed that deletion of the ENA transporters, leaving presumably only NHA1 to export lithium, produced yeast cells many times more sensitive to lithium than the wild type (Zörgö et al. 2013). The wide range of lithium sensitivity in yeast strains observed by Petrezselyova et al. (Petrezselyova et al. 2010) might be explained by different patterns of sodium transporter expression and density at the cell membrane. Based on BLAST against the human proteins, the yeast PMR/ENA family members are part of a larger family shared with humans of cation-transport ATPases. NHA1, however, does not appear to be closely related to any of the human sodium-proton antiporters, neither the human NHE family (Orlowski and Grinstein 2004) nor to human NHA2 (Fuster et al. 2008).

Because of the high degree of orthology between mammalian lithium-sensitive magnesium-dependent enzymes and their yeast counterparts, it is reasonable to expect that the lithium sensitivity of biochemical pathways will be found to be based on the antagonism between Li+ and Mg++. Experimentally this is so far supported. The lithium inhibition on phosphoglucomutase can be relieved by high magnesium concentrations (Masuda et al. 2001). Referring to a cellular phenotype, yeast gametogenesis is promoted by Mg++ (Suizu et al. 1994) but inhibited by Li+ (Asensio et al. 1976).

In summary, in yeast as in mammalian cells, lithium–sodium competition for membrane transport modulates the intracellular lithium concentration, and lithium-magnesium competition for binding sites in enzymes modulates the biochemical phenotype. It appears that the yeast is a useful model system for understanding lithium effects in eukaryotes.

## Origins and Evolution of Lithium in Biology

It is a prevalent view that a precursor to the emergence of biochemistry as we know it consisted of networks of replicating RNA molecules (Higgs and Lehman 2015). An essential component of the RNA world would have been the oligomerization of RNA molecules into long enough molecules to have distinctive identities, one from the other. In one possible scenario this was catalyzed on clay surfaces with small ions such as lithium and sodium as essential co-factors (Joshi and Aldersley 2013; Ferris 2005).

Only a few studies have been done on either the organismic or the biochemical response of prokaryotes to lithium. One such study found that gram-positive bacteria can take up much more lithium from an aqueous medium than yeast or other fungi, so we know there are pathways for entry (Tsuruta 2005).

In the absence of direct experimental data, sequence homologies and orthologies can at least provide plausible hypotheses about the extent to which eukaryotic lithium transport mechanisms and lithium-sensitive enzymes are also present in bacteria. Presumably significant paths of lithium entry into bacteria are by sodium channels (Koishi et al. 2004) and pentameric ligand-gated channels (Tasneem et al. 2004) orthologous to the corresponding metazoan channels that have been shown to be Li+-permeable (Richelson 1977; Krishnamurthy et al. 2009; Grosman, Claudio 2016. Personal communication based on unpublished observations). Based on a sequence search for orthologs in the Uniprot database (unpublished), it appears that the NHA1 proton-sodium antiporter that extrudes lithium from the yeast cell has orthologs in bacteria, so this may be a common path for lithium to leave. The sodium-proton antiporter that enables export of lithium in metazoan cells (Busch et al. 1995), has numerous orthologs in both bacteria and archaea, with the transport function verified experimentally (Rahav-Manor et al. 1992). On the other hand, reciprocal BLAST by us (unpublished) finds that the closest bacterial homologs to the yeast ENA sodium transporter are orthologous to yeast calcium transporters, so it may be that this mechanism for lithium export is uncommon or lacking in bacteria.

Orthologs to the magnesium-dependent lithium-sensitive eukaryotic protein kinases are abundant in bacteria and archaea (Leonard et al. 1998). Bacteria also contain members of the magnesium-dependent lithium-sensitive phosphomonoesterase family (York et al. 1995).

It appears that lithium has had a significant effect on first molecular and then biological evolution, and that major elements of lithium transport and modulation of biochemical function are shared across all classes of living systems.

## Summary

- Many aspects of the distribution of lithium within cells and extracellular fluid are due to competition of lithium with sodium for sites in transporters and channels that are primarily sodium-selective. Much remains to be learned about the details of this competition.
- Many biochemical effects of lithium are due to competition of lithium with magnesium for sites in biomolecules that are primarily magnesium-selective. Much remains to be learned about the details of this competition. Much also remains to be learned about how the multiple molecular targets of lithium, and the many more substrates of lithium targets, interact to modulate the integrated behavior of cells.
- Few studies have been done on the effect of lithium on membranes. Those studies suggest significant effects of lithium on local membrane structure and on distribution of signaling proteins between rafts and fluid regions of membranes.
- Levels of ingested lithium appear to have profound effects on human well-being across the spectrum of normal health, and susceptibility to important diseases, such as mood disorders, neurodegenerative disease, and cancer. Much remains to be learned and understood about the biochemistry underlying the full range of these effects and the implications of that biochemistry for nutrition and medicine. Such explorations will be aided by the fact that the many and widely varying effects of lithium seem to be caused by only a small number of underlying biophysical mechanisms. On the other hand, the widespread nature of the effects requires the application of the tools and approach of systems biology, which has so far been done only in a very preliminary way.

## Electronic supplementary material

Below is the link to the electronic supplementary material.

Supplementary material 1 (DOCX 115 kb)
