# Supplementary material for: Towards a Unified Understanding of Lithium Action in Basic Biology and its Significance for Applied Biology
Source: J Membr Biol. 2017 Nov 10;250(6):587–604. doi: 10.1007/s00232-017-9998-2 (PMC5696506; doi:10.1007/s00232-017-9998-2)
Supplement: Supplementary file 1 — Supplementary material 1 (DOCX 115 kb) [file 232_2017_9998_MOESM1_ESM.docx]

**Supplementary Material**

To replicate the search resulting in Figure 2 and Table 1, go to the web site of the STRING database, <http://string-db.org/>

In the “search” box put gsk3b and homo sapiens, as shown below, and click the search button:


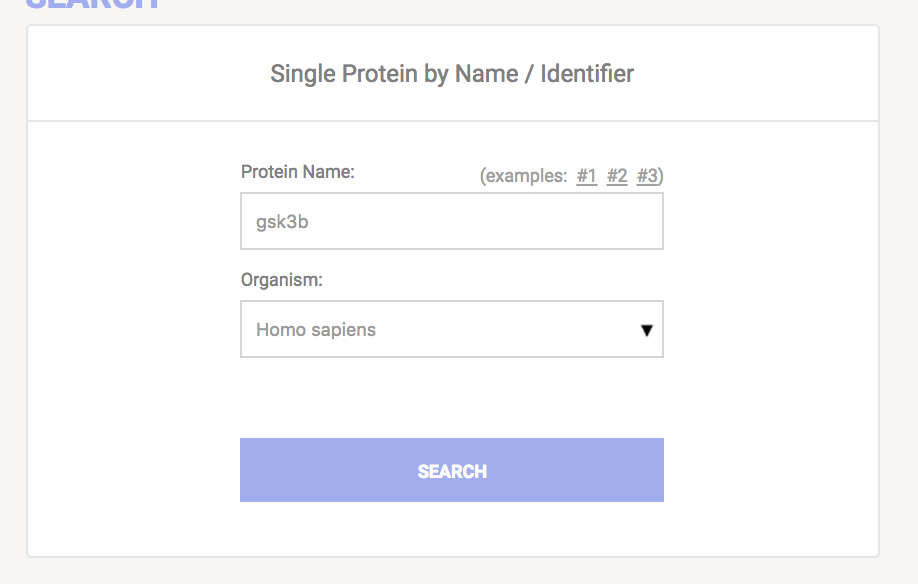


In the next screen, press the “continue” button.

In the next screen, shown below, press the “data settings” button. Within data settings, set the maximum number of elements in the first shell to 300 and the confidence level to 0.7 Then press the “update settings” button.

This will produce the visualization of Figure 2 (with live links to the gene names), the information in Table 1, and links to much more relevant information.
